# Baseline neutrophil–lymphocyte ratio can be associated with hematoma expansion in patients with intracerebral hemorrhage: a retrospective observational study

**DOI:** 10.1186/s12868-022-00705-z

**Published:** 2022-03-25

**Authors:** Ehsan Alimohammadi, Seyed Reza Bagheri, Parand Mardanpour, Farid Moradi, Fatemeh Arjmandnia, Narges Esmaeili

**Affiliations:** 1 Department of Neurosurgery, Imam Reza Hospital, Kermanshah University of Medical Sciences, Kermanshah, Iran; 2 Clinical Research Development Center, Imam Reza Hospital, Kermanshah University of Medical Sciences, Kermanshah, Iran; 3 Department of Anesthesiology, Kermanshah University of Medical Sciences, Kermanshah, Iran; 4 Kermanshah University of Medical Sciences, Kermanshah, Iran

**Keywords:** Neutrophil-to-lymphocyte ratio, Intracerebral hemorrhage, Hematoma expansion, Glasgow Coma Scale

## Abstract

**Background:**

Hematoma expansion can be related to increased mortality and poor clinical outcomes in patients with intracerebral hemorrhage (ICH). So, early identification and prevention of hematoma expansion can be considered as an important therapeutic aim. This study aimed to evaluate the hypothesis that the neutrophil to lymphocyte ratio (NLR) is associated with hematoma expansion in ICH patients.

**Methods:**

We retrospectively evaluated the clinical data of a total of 221 patients with ICH who were treated in our department between April 2018 and April 2021. The demographic, clinical, radiological, and laboratory test data including the NLR upon admission were investigated. A binary logistic regression analysis was used to assess the independent associations between different variables and hematoma expansion.

**Results:**

A total of 221 patients with ICH were included. There were 122 (55.2%) males and 99 (44.8%) females. The mean age (years) at admission was 66.43 ± 8.28.

The hematoma expansion occurred in 57 (25.8%) cases. The results of the multivariate analysis showed that hematoma volume at baseline (OR, 3.12; 95% CI 1.78–5.02; P < 0.001), admission systolic blood pressure (OR, 2.87; 95% CI 1.79–4.34; P = 0.013), Glasgow Coma Scale (GCS) (OR, 1.94; 95% CI 1.45–2.93; P = 0.020), and NLR (OR, 1.74; 95% CI 1.16–2.60; P = 0.032) were correlated with hematoma expansion in these patients.

**Conclusions:**

Our findings suggest that NLR can be a predictor of hematoma expansion in patients with ICH. This cost-effective and easily available biomarker could be used to early prediction of hematoma expansion in these patients.

## Introduction

Intracerebral hemorrhage (ICH) is one of the most common causes of morbidity and mortality throughout the world with an estimated 35–52% rate of 30-day mortality [1, 2].

Hematoma expansion occurs in approximately 30% of ICH cases within the first 24 h [1, 3]. It has been demonstrated that hematoma expansion is related to increased mortality and poor clinical outcomes [4, 5]. As a result, early identification and prevention of hematoma expansion can be considered an important therapeutic aim.

Several factors have been known as the predictors of hematoma expansion including the size and location of the hematoma, elevated systolic blood pressure, the presence of coagulopathy, and the presence of systemic inflammatory response syndrome (SIRS) during hospitalization [6–8].

Some studies have suggested absolute and differential leukocyte counts as a marker for central nervous system inflammation [9]. The inflammatory response can lead to a series of neurochemical cascade events including alteration in cerebral blood flow, breakdown of the blood–brain barrier, dysfunction of brain tissue metabolism, and cell damage [10, 11]. In a retrospective study, Chen et.al investigated the predictive value of NLR for the prognosis of patients with severe traumatic brain injury (TBI). Their results showed that the baseline NLR was significantly higher in the unfavorable outcome group than in the favorable outcome group and the higher NLR was related to an unfavorable outcome. It has been shown that elevated neutrophil–lymphocyte ratio (NLR) in patients with ICH can be associated with subsequent neurologic deterioration, higher 30-day mortality, and stroke severity [9, 12].

The present study aimed to evaluate the relationship between NLR and hematoma expansion.

## Methods

All consecutive patients with spontaneous ICH presenting to Imam Reza hospital, Kermanshah, Iran from April 2018 and April 2021 were investigated retrospectively. We included all ICH patients who had a primary spontaneous ICH and at least two head CTs obtained within the first 24 h of admission. The exclusion criteria of the study were: age less than 18 at admission, secondary causes of ICH (i.e., trauma, aneurysms, tumors, and arteriovenous malformations), history of anticoagulant medications, conditions with associated leukocytosis, such as infection and hematologic malignancies. This study was approved by the Scientific Research Board of the Kermanshah University of Medical Sciences.

The demographic, clinical, radiological, and laboratory test data were extracted from hospital medical records.

We determined the location of hematoma according to the initial brain CT scan of all patients and divided the location of the hematoma into four categories including lobar, deep, cerebellar, and brain stem.

The hematoma volume was calculated according to the ellipsoid formula (4/3 π a × b × c), where a, b, and c represents the respective radii in 3-dimensional neuroimaging [13].

Hematoma expansion was defined as relative enlargement > 33% or absolute growth > 6 mL [4].

We evaluated the clinical outcome at the time of hospital discharge using the Glasgow Outcome Scale (GOS) [14]. The GOS measures global functioning with five outcome categories: (1) death, (2) persistent vegetative state, (3) severe disability, (4) moderate disability, and (5) good recovery. We classified the GOS groups in binary categories: favorable (GOS 4, 5) and unfavorable (GOS 1, 2, 3).

Blood sampling was attended on admission. Neutrophil and lymphocyte counts were collected based on the peripheral hemogram which was evaluated using venous blood samples by an automated blood counter (XN-10, Sysmex Inc., Japan).

We calculated NLR by dividing the absolute neutrophil count by the lymphocyte count.

### Statistical analysis

Data are presented as mean ± standard deviation. The independent t-test, the Chi-square test, and Fisher’s exact test were used to compare different variables between the groups. A binary logistic regression analysis was used to assess the independent associations between different variables and hematoma expansion. The data analysis was performed using the SPSS 21 software (SPSS Inc. Chicago, Illinois). P values < 0.05 were considered as the significant level.

## Results

We investigated a total of 221 patients with ICH. There were 122 (55.2%) males and 99 (44.8%) females. The mean age (years) at admission was 66.43 ± 8.28. The hematoma expansion occurred in 57 (25.8%) cases. The descriptive characteristics of the sample are presented in Tables 1 and 2.

**Table 1:** Frequency and frequency percent of the variables

Variable	Frequency	Frequency percent
Hematoma expansion		
Yes	57	25.8
No	164	74.2
Gender		
Male	122	55.2
Female	99	44.8
Hypertension		
Yes	133	60.2
No	88	39.8
Diabetes		
Yes	60	27.1
No	161	72.9
Smoking		
Yes	63	28.5
No	158	71.5
Hematoma location		
Lobar	71	32.1
Deep	99	44.8
Cerebellar	33	14.9
Brain stem	18	8.1
GOS		
Death	46	20.8
Vegetative state	25	11.3
Severe disability	49	22.2
Moderate disability	61	27.6
Good recovery	40	18.1
Need for surgery		
Yes	64	29.0
No	157	71.0
Intera-ventricular hemorrhage		
Yes	48	21.7
No	173	78.3
Hydrocephalus		
Yes	31	14.0
No	190	86.0

*GOS* Glasgow Outcome Scale

**Table 2:** Mean and standard deviation of quantitative variables

Variable	Mean (SD)
Age (year)	66. 43 (8.29)
GCS	8.94 (1.73)
Hospital stay (day)	17.21 (7.16)
Hematoma volume at baseline (mL)	14.57 (5.61)
Hematoma volume at 24 h (mL)	16.39 (6.11)
Time to baseline CT scan, h	4.67 (1.32)
Time to 24-h CT scan, h	23.8 (1.71)
Admission systolic blood pressure (mmHg)	157.39 (7.21)
Admission diastolic blood pressure (mmHg)	89.32 (5.13)
Baseline white blood cell count cells/mm^3^	9360 (5404)
Neutrophil count, ×10^9^ cells/L	9.73 (1.41)
Lymphocyte count, ×10^9^ cells/L	1.63 (1.01)
Baseline NLR (no units)	7.43 (1.38)
Platelet count cells/mm^3^	205,087 (9004)
Admission prothrombin time	13.79 (1.12)
Admission partial thromboplastin time	33.13 (3.12)
Admission INR	1.2 (0.38)

*GCS* Glasgow Coma Scale, *NLR* neutrophil to lymphocyte ratio, *INR* international normalized ratio

Patients with hematoma expansion had a worse outcome in comparison with those without hematoma expansion (p < 0.05; Table 3). As shown in Table 3 the need for surgery was higher in the patients in the hematoma expansion group compared to cases in the non-hematoma expansion group (p < 0.05; Table 3).

**Table 3:** Comparing two groups (hematoma expansion group–non hematoma expansion group) in term of qualitative variables

Variable	Hematoma expansion		Statistical test
	Yes (n = 57)	No (n = 164)	
Gender			
Male	33 (27.7)	89 (72.3)	P = 0.643
Female	24 (24.2)	75 (76.8)	
Hypertension			
Yes	31 (23.3)	102 (76.69)	P = 0.321
No	26 (29.5)	62 (70.5)	
Diabetes			
Yes	16 (26.6)	44 (73.4)	P = 0.508
No	41 (25.4)	120 (74.5)	
Smoking			
Yes	14 (22.2)	49 (77.8)	P = 0.754
No	43 (27.2)	115 (72.8)	
Hematoma location			
Lobar	28 (28.3)	71 (71.7)	P = 0.866
Deep	17 (23.9)	54 (76.1)	
Cerebellar	7 (21.2)	26 (78.8)	
Brain stem	5 (27.7)	13 (72.2)	
GOS			
Unfavorable outcome			
Death	14 (30.4)	32 (69.6)	**P = 0.017**
Vegetative state	7 (28)	18 (72)	
Sever disability	13 (26.5)	36 (73.5)	
Favorable outcome			
Moderate disability	14 (22.9)	47 (77.1)	
Good recovery	9 (22.5)	31 (77.5)	
Need for surgery			
Yes	28 (43.7)	36 (56.3)	**P = 0.011**
No	29 (18.4)	128 (81.6)	
Intera-ventricular hemorrhage			
Yes	12 (25.0)	36 (75.0)	P = 0.210
No	45 (26.0)	128 (74.0)	
Hydrocephalus			
Yes	7 (22.5)	24 (77.5)	P = 0.625
No	50 (26.3)	140 (73.7)	

Bold indicates p < 0.05

*GOS* Glasgow Outcome Scale

According to the univariate analysis, GCS, hematoma volume at baseline, admission systolic blood pressure, the baseline neutrophil count, and the baseline NLR were associated with hematoma expansion in patients with ICH (p < 0.05; Tables 3, 4).

**Table 4:** Comparing two groups (hematoma expansion group–nonhematoma expansion group) in terms of quantitative variables

Variable	Hematoma expansion		Hematoma expansion
	Yes (n = 57)	Yes (n = 57)	
Age (year)	64.37 (4.36)	65.18 (4.42)	P = 0.437
GCS	6.31 (1.23)	9.61 (1.95)	**P = 0.021**
Hospital stay (day)	21.31 (5.42)	13.87 (6.21)	**P = 0.033**
Hematoma volume at baseline (mL)	23.16 (6.41)	15.32 (5.25)	**P = 0.008**
Admission systolic blood pressure (mmHg)	173.56 (9.49)	152.11 (7.81)	**P = 0.024**
Admission diastolic blood pressure (mmHg)	89.21 (6.41)	87.56 (6.01)	P = 0.435
Baseline white blood cell count cells/mm^3^	10,353 (4624)	9673 (4714)	P = 0.321
Neutrophil count, ×10^9^ cells/L	9.72 (1.37)	8.31 (1.36)	**P = 0.023**
Lymphocyte count, ×10^9^ cells/L	1.27 (1.03)	1.75 (1.02)	P = 0.071
Baseline NLR (no units)	7.65 (1.38)	4.74 (1.38)	**P = 0.012**
Platelet count cells/mm^3^	193,214 (8760)	207,167 (9124)	P = 0.454
Admission prothrombin time	13.14 (1.12)	13.83 (1.13)	P = 0.651
Admission partial thromboplastin time	33.54 (3.02)	35.11 (3.11)	P = 0.211
Admission INR	1.3 (0.37)	1.2 (0.36)	P = 0.304

Bold indicates p < 0.05

*GCS* Glasgow Coma Scale, *NLR* neutrophil to lymphocyte ratio, *INR* international normalized ratio

The results of the multivariate analysis showed that hematoma volume at baseline (OR, 3.12; 95% CI 1.78–5.02; P < 0.001), admission systolic blood pressure (OR, 2.87; 95% CI 1.79–4.34; P = 0.013), GCS (OR, 1.94; 95% CI 1.45–2.93; P = 0.020), and NLR (OR, 1.74; 95% CI 1.16–2.60; P = 0.032) were correlated with hematoma expansion in these patients (Table 5).

**Table 5:** Binary logistic regression analysis of hematoma expansion after intracerebral hemorrhage

Variables	Odds ratio	95% CI	P-value
Hematoma volume at baseline (mL)	3.12	1.78–5.02	**P < 0.001**
Admission systolic blood pressure (mmHg)	2.87	1.79–4.34	**P = 0.013**
GCS	1.94	1.45–2.93	**P = 0.020**
Neutrophil count	1.26	0.80–1.63	P = 0.271
Baseline NLR	1.74	1.16–2.60	**P = 0.032**

Bold indicates p < 0.05

*GCS* Glasgow Coma Scale, *NLR* neutrophil to lymphocyte ratio

## Discussion

The results of the present study show that baseline NLR can be correlated with 24-h hematoma expansion after ICH. It has been demonstrated that the inflammatory response after ICH can lead to peripheral leukocytosis. The hemorrhage leads to microglial activation and as a result secrete cytokines and chemokines that can promote leukocyte infiltration within hours.

Some studies showed that astrocytes shed extracellular vesicles which regulate peripheral leukocyte response in response to brain inflammation.

The inflammatory response can result in a series of neurochemical cascade events including alteration in cerebral blood flow, breakdown of the blood–brain barrier, dysfunction of brain tissue metabolism, and cell damage [10, 11]. The NLR is considered as a nonspecific marker of systemic inflammation [15]. Elevated NLR has been found to be related to poor prognosis in patients with ICH and those with traumatic brain injury [16–18]. Jamali et al. in their retrospective study found that an NLR > 12.5 at admission can predict higher mortality in patients with aneurysmal subarachnoid hemorrhage [19]. In another study, Chen et al. evaluated the relationship between peak NLR and clinical outcomes of patients with severe TBI. They reported that peak NLR can be a predictor for unfavorable outcomes after severe TBI [16].

Neutrophils are the major component of the innate immune system that play a major role in mediating inflammation-induced injury [20–22]. It has been demonstrated that, more than inflammation-related cytokines, neutrophils also contain angiogenic and neurotrophic factors [11, 23, 24].

Moreover, neutrophils are associated with vascular dysfunction that leads to cerebral hypoperfusion [25]. The hypoperfusion may lead to an increase in the interactions of neutrophils with blood vessels by inducing the expression of l-selectin and intercellular adhesion molecule 1 in endothelial cells [26]. So, neutrophils may affect microcirculation rheology as well as the sustained pressure of the microvasculature [27]. Neutrophils could alter cerebral blood flow by forming pseudopods and adhering to the endothelium and platelets [28].

As mentioned above, the inflammatory response could be associated with hematoma expansion. So, the peripheral leukocyte counts may help in predicting hematoma expansion after ICH.

## Limitations

The present study has several limitations. It is a retrospective single-center study with relatively small sample size. Bias in terms of data selection and analysis due to the retrospective nature of the study may be considered as another limitation of our study.

Meanwhile, we evaluated hematoma expansion only up to the first 24 h, whereas it is known that hematoma expansion may evolve beyond this time frame [29]. Finally, we did not have data on body temperature and osmotherapy, both may be related to hematoma expansion [30].

## Conclusions

Our findings suggest that NLR can be a predictor of hematoma expansion in patients with ICH. Further studies are warranted to understand the association between NLR and hematoma expansion. However, this cost-effective and easily available biomarker, along with previously established variables, could be used to early prediction of hematoma expansion in patients with ICH.

## Data Availability

The datasets generated and/or analysed during the current study are not publicly available due them containing information that could compromise research participant privacy/consent but are available from the corresponding author on reasonable request.

## Abbreviations

NLR: Neutrophil to lymphocyte ratio
ICH: Intracerebral hemorrhage
GOS: Glasgow Outcome Scale
SIRS: Systemic inflammatory response syndrome
